# The Treatment of Hip Joint Disease

**Published:** 1893-01-28

**Authors:** 


### BRISTOL CHILDREN'S HOSPITAL.

### The Treatment of Hip Joint Disease.

When patients are admitted with hip joint disease in an early stage, *i.e.*, before there are signs of abscess about the joint, they are kept in bed with extension on the limb and a long splint on the other side to steady the body. The foot of the bed is raised so as to provide counter-extension by the body tending to slide in the opposite direction to that in which the weight is pulling. When flexion is extreme the extension is applied with the child lying on the side until the flexion is very largely overcome. In some very early cases all the symptoms subside after some weeks of this treatment, free and painless movement at the joint returning, but such cases are not allowed at once to get about again without fixing the joint (for fear of recurrence of the trouble), but are sent out in a Thomas splint.

A child will often be admitted with a distinct history of night starting, which does not recur after the commencement of treatment by rest and extension. Of course extension does not pull the joint surfaces apart; it acts by steadying the joint, preventing reflex spasm of the muscles, pressing and jarring the joint surfaces together. It is this sudden jarring of the joint surfaces together just as the child drops off to sleep, when the contracted muscles guarding the joint from movement are off their guard, which causes the "night starting" so painful in cases of hip diseases. The extension should always be made from above the knee, otherwise the knee joint gets stretched, and the bony points about the joint should be protected by a bandage from the pressure of the strips of plaster as they pass over it at the sides.

Notwithstanding rest and extension, in many cases abscess forms in the neighbourhood of the joint, and when this happens the joint is as a rule excised. This is done by the posterior incision, which is preferred to the anterior, as giving greater facilities of exploring the joint and removing the end of the femur. The trochanter is always removed as well as the head of the bone, as in the greater number of cases it is found diseased, and as this cannot be determined before removal, it is always excised together with the head. The bone is divided with Adam's saw, and the head and trochanter removed with sequestrum forceps. Often a sequestrum is found in the head or neck. The operation is performed antiseptically, and joint syringed out after removal of the bone with an antiseptic solution. The child is always put up at the time of the operation in Bryant's hip splint, which is really a combination of two long splints joined together in a common frame, extension being made by means of an elastic cord, making traction on the foot piece.

If cases do not do well after excision they are sent to the seaside in a Thomas splint, to a home where the dressing of the sinus can be carefully attended to. Sometimes the joint is explored for fresh sequestra from the acetabulum, and carious bone there is scraped away, or the disease is found to be extending down the shaft of the femur. If, after excision, with unhealed, freely suppurating sinuses, change to the seaside does no good, and the child is markedly going downhill, amputation at the hip joint may enable the child to recover from the exhaustion of the disease, even though the carious acetabulum could not be removed. The removal of the end of the femur (from which the head has before been removed) from this cavity, and the ability to more thoroughly scrape it out, perhaps gives it a better chance of recovery.

But we look forward to more hopeful days of hip surgery. In Barker's hands, his method of excision has given most hopeful results. If such results can be obtained by other men, these chronic sinuses after excision will be things of the past, and we shall no longer hesitate whether we ought to excise or merely open a hip abscess. Barker's method and his results are described in a paper in The Hospital in the early part of last year.

